# Selection and characterization of *Bacillus thuringiensis* strains from northwestern Himalayas toxic against *Helicoverpa armigera*

**DOI:** 10.1002/mbo3.484

**Published:** 2017-10-18

**Authors:** Showkat A. Lone, Abdul Malik, Jasdeep C. Padaria

**Affiliations:** ^1^ Department of Agricultural Microbiology Faculty of Agricultural Sciences Aligarh Muslim University Aligarh India; ^2^ Biotechnology and Climate Change Group ICAR‐National Research Centre on Plant Biotechnology (NRCPB) New Delhi India

**Keywords:** *Bacillus thuringiensis*, Bioassay, Cry proteins, *Helicoverpa armigera*, LC_50_, PCR

## Abstract

In this study, we present the selection and the characterization of *Bacillus thuringiensis* (*Bt*) strains with respect to their *cry*/*cyt* gene content and toxicity evaluation toward one of the most important polyphagous lepidopteran pest, *Helicoverpa armigera*. Fifty‐six *Bt* isolates were obtained from 10 different regions of northwestern Himalayas, recording a total *B. thuringiensis* index of 0.62. Scanning electron microscopy revealed presence of bipyramidal, spherical, flat and irregular crystal shapes; SDS‐PAGE analysis of spore‐crystal mixtures showed the prominence of 130, 70, and 100 kDa protein bands in majority of the isolates; PCR analysis with primers for eight *cry* and *cyt* gene families and 13 *cry* gene subfamilies resulted in isolates showing different combinations of insecticidal genes. Strains containing *cry*1 were the most abundant (57.1%) followed by *cyt*2 (46.42%), *cry*11 (37.5%), *cry*2 (28.57%), *cry*4 (21.42%), *cyt*1 (19.64%), *cry*3 (8.9%), and *cry*7, 8 (7.14%). A total of 30.35% of the strains did not amplify with any of the primers used in this study. Median lethal concentration 50 (LC_50_) estimates of spore‐crystal mixtures of *Bt*‐JK12, 17, 22, 48, and 72 against second instar larvae of *H. armigera* was observed to be 184.62, 275.39, 256.29, 259.93 μg ml^−1^, respectively. *B. thuringiensis* presents great diversity with respect to the presence of crystal protein encoding genes and insecticidal activity. Four putative toxic isolates identified in this study have potential application in insect pest control. *B. thuringiensis* isolate JK12 exhibited higher toxicity against *H. armigera* than that of *B. thuringiensis* HD1, hence can be commercially exploited to control insect pest for sustainable crop production. The results of this study confirm the significance of continuous exploration of new *Bt* stains from different ecological regions of the world.

## 1 Introduction

The escalating public concern, stringent environmental regulations, and build‐up of resistant insect populations to synthetic pesticides have led to an increased interest in alternative eco‐friendly pest control strategies. One of the most successful substitutes to the manmade pesticides is the use of entomopathogenic bacterium, *Bacillus thuringiensis* (*Bt*). The entomopathogenic potential of *Bt* is primarily due to its ability to synthesize during sporulation, the crystalline proteins, Cry and Cyt, encoded by *cry* and *cyt* genes, respectively (Schnepf et al., 1998). Up to March 2017, 74 classes of Cry proteins (Cry1 to Cry74) and three classes of Cyt proteins (Cyt1‐Cyt3) have been designated based on their amino acid sequence homology (Crickmore et al., 2017). These toxins are highly specific in action, harmless to humans and other vertebrates and are biodegradable. The reasons for increased acceptability of *Bt* over synthetic insecticides are due to the nonselective deleterious effects of chemicals (Eriksson & Wiktelius, 2011) and the emergence of resistance in insect pests against the synthetic insecticides (Ahmad, Sayyed, & Saleem, 2008). For the said reasons, continuous efforts are being made to isolate novel *B. thuringiensis* strains with distinctive host range or higher toxicity potential.

The presence of *Bt* has been extensively studied in different ecological habitats such as soil, stored product dust, dead insects, food grains, phyllosphere, and aquatic environments (Ben‐Dov et al., 1997; Bravo et al., 1998; Martin & Travers, 1989). Diversity of *B. thuringiensis* and their *cry* genes from different regions of India have been studied earlier (Kaur & Singh, 2000; Patel, Purani, & Ingle, 2013). However, distribution and diversity of *B. thuringiensis* and their *cry* and *cyt* genes in *B. thuringiensis* isolates from northwestern Himalayan region has not yet been approached in detail (Yadav, Sachan, Verma, & Saxena, 2016). The variations in topographical features along longitude, latitude, and altitude of the region create climatic variations resulting in unique and rich biodiversity (Ray, Doshi, Alag, & Sreedhar, 2011), thereby making the northwestern Himalayan region a critical biodiversity hotspot of the world. These distinctive features and diversity of insects in the region provide an opportunity for prospecting novel *B. thuringiensis* strains with novel combinations of crystalline protein coding genes having wide insecticidal spectrum. Various methods such as polymerase chain reaction (PCR), Southern blotting, protein profiling, serotyping, and bioassay have been employed for the characterization of *B. thuringiensis* strains and their crystalline protein‐encoding genes; among all, PCR‐based methods have proven to be the most efficient in terms of rapidity in large‐scale screening programs (Porcar & Juarez‐Perez, 2003). In this study, a combination of three methods *viz*. PCR, protein profiling, and insect bioassay was used to characterize the *B. thuringiensis* strain collection.

The aim of this study was to isolate potentially toxic *B. thuringiensis* strains from northwestern Himalayas, which represents one of the hotspots of biodiversity. The strains were characterized based on the presence of protein crystals, SDS‐PAGE analysis, and PCR analysis to identify different *cry* and *cyt* gene combinations and toxicity screening against *Helicoverpa armigera*. The type of *cry/cyt* genes present in a particular strain defines their host range, so the strains harboring several types of *cry*/*cyt* can be predicted to have a wider spectrum or additive activity. Such studies are useful in understanding the distribution of *cry* and *cyt* genes and may lead to identification of broad‐spectrum and effective isolates for the control of various insect pests.

## 2 Materials and Methods

### 2.1 Sample collection, isolation of *B. thuringiensis*, and crystal characterization

The 89 strains analyzed in this study were isolated from 207 soil samples collected from three zones consisting of 10 locations of northwestern Himalayas. Sites with no history of use of *Bt* or its products were selected (Table 1). Isolation of *Bt* was done by enrichment using acetate selection as described by Travers, Martin, and Reichelderfer (1987). Following acetate selection, isolates that tested positive for growth on Luria medium amended with penicillin at a concentration of 10 μg ml^−1^ were examined for the presence of parasporal crystals (Ohba & Aizaw, 1986; Yang, Wu, Wang, & Xie, 2000). Crystals were characterized using phase contrast microscopy and the shape of crystals was confirmed by scanning electron microscopy (Lone, Yadav, Malik, & Padaria, 2016). *Bt* index was calculated as described by Baig, Bukhari, and Shakoori (2010).

**Table 1:** Distribution of sample collection sites and recovery of *Bacillus thuringiensis*

Geographical areas (northwestern Himalayas)	No. of samples examined	No. of colonies obtained	No. of *Bacillus* like isolates	no. of crystalliferous colonies	Bt index^a^
Agricultural land					
Lolab	23	132	14	9	0.64
Aru	25	118	13	11	0.84
Saripara	15	126	12	8	0.66
Urusa	23	118	9	3	0.33
Venkra	17	115	8	4	0.50
Forests					
Brarinar	28	136	8	8	1.00
Afferwat	22	122	7	5	0.71
Sonmarg	27	114	4	3	0.75
Zanskar	22	128	6	3	0.50
Lake sediments					
Pangkong	5	92	8	2	0.25
Total	207	1201	89	56	0.62

a Ratio between number of *B. thuringiensis* colonies and total number of sporulating colonies examined.

### 2.2 Standard bacterial strains

Four standard *B. thuringiensis* (*Bt*) strains; *Bt*. subsp. *kurstaki* (BGSC 4D1), *Bt*. subsp. *aizawai* (BGSC 4J3), *Bt*. subsp. *israelensis* (BGSC 4Q1), and *Bt*. subsp. *morrisoni biovar. tenebrionis* (BGSC 4AA1) were kindly provided by Dr. Daniel R. Zeigler from the *Bacillus* Genetic Stock Center (Ohio, USA). These strains were used as reference wherever required.

### 2.3 PCR analysis of crystalline protein genes

DNA template was prepared by lysis precipitation (Rampersad & Ammons, 2005). All primer pairs used for detection of *cry* and *cyt* gene families (*cry*1, *cry*2, *cry*3, *cry*4, *cry*7, *cry*8, *cry*11, *cyt*1, *cyt*2) have already been documented (Juarez‐Perez, Ferrandis, and Frutos, 1997; Ben‐Dov et al., 1997; Ibarra et al., 2003). Table S1 shows sequences of all primers used in the study, their source, expected amplicon sizes, optimal annealing temperatures and respective positive control DNA used. For detection of crystalline protein gene combinations, all the native *B. thuringiensis* isolates were subjected to PCR analysis. PCR was performed in an Eppendorf Mastercycler and amplification conditions were as follows: an initial denaturation at 94°C for 5 min, followed by 30 cycles of denaturation at 94°C for 60 s, annealing at variable temperature depending on the primer pair (Table S1) for 60 s, and extension at 72°C for 60 to 90 s (depending on amplicon expected size), and a final extension step at 72°C for 10 min. Isolates showing the presence of lepidopteran‐specific *cry*1 and *cry*2 genes were further screened using *cry*1 (*cry*1Aa, *cry*1Ab, *cry*1Ac, *cry*1Ad, *cry*1B, *cry*1C, *cry*1D, *cry*1E, *cry*1F, *cry*1G) and *cry*2 (*cry*2Aa, *cry*2Ab, *cry*2Ac) subfamily primers, respectively.

### 2.4 Protein profiling of *B. thuringiensis* isolates

The purpose of protein analysis was to select isolates exhibiting distinct protein profiles and to characterize each isolate of *B. thuringiensis* collection. All the native *B. thuringiensis* isolates and reference strain (*Bt* HD1) were grown in T3 broth for 72 hr at 30°C, the cultures were pelleted by centrifugation at 12,000*g* at 4°C for 5 min and the pellets were washed once with sterile distilled water followed by washing with 1 mML^−1^ NaCl containing 5 mML^−1^ EDTA. Protein composition of spore‐crystal mixtures was analyzed by sodium dodecyl sulfate polyacrylamide gel electrophoresis (SDS‐PAGE), as previously described (Laemmli, 1970).

### 2.5 *Helicoverpa* culture

*Helicoverpa armigera* larvae were initially maintained on natural diet consisting of chick pea leaves and pods up to second generation and the subsequent generations were reared on artificial diet consisting of gL^−1^/mlL^−1^ pollen (15), soy flour (86), wheat germ (60), yeast extract (50), ascorbic acid (3), nipagin (3), sorbic acid (1), sunflower oil (5), and agar (12) in water. During oviposition, *H. armigera* adults were provided with 5% (w/v) honey solution. Larvae were maintained up to 10 generations before proceeding with bioassay experiments. Both rearing and bioassays were carried out at 25°C, 50 ± 10% relative humidity, with 14 hr photoperiod (Teakle & Jensen, 1985).

### 2.6 Bioassay

Mass screening of *B. thuringiensis* collection for toxicity against *H. armigera* was performed using relatively higher concentration (1 mg ml^−1^) of spore‐crystal mixtures. All the *B. thuringiensis* strains were cultured in 50 ml CCY medium (Stewart, Johnstone, Hagelberg, & Ellar, 1981) for 72–96 hr at 30°C, optimal crystal formation was checked by phase contrast microscopy. The cells were harvested by centrifugation at 9,000*g* for 10 min, the pellet obtained was washed twice with cold 1mol/L NaCl and twice with cold autoclaved distilled water. The wet and dry weight of final pellet (spore‐crystal, mixture) was determined and stored at −20°C until bioassay (Baig et al., 2010). The density of bacterial doses was optimized on *cry*1Ac harboring positive control strain of *Bt* HD‐1. All steps were carried out at 4°C to limit proteolysis. Second instar larvae of *H. armigera* were used for bioassay by diet incorporation method (Song et al., 2003). The conditions for bioassay were as mentioned above, 30 larvae per treatment were used. In the control group, the culture was substituted with sterile distilled water. Mortality was recorded after 7 days, larvae were scored as dead if they failed to respond to gentle probing, *Bt* HD1 was used as positive control for bioassays. Isolates showing 96%–100% mortality in the preliminary bioassay screening were subjected to more precise bioassays using six concentrations (50 μg ml^−1^ to 800 μg ml^−1^) of spore‐crystal mixtures. Each dose was thoroughly mixed in 3 ml of artificial diet prior to solidification and poured into wells. Twenty‐four second instar larvae per treatment were used, and the experiment was set in triplicate, making total number of larvae tested per treatment to 72. The mortality observed was corrected to control mortality by Abbott's formula (Abbott, 1925). The median lethal concentration 50 (LC_50_) was determined by probit analysis using the software SPSS version 19.0. for windows. In order to rule out toxicity as a function of β‐exotoxin production, the supernatant from the toxic isolates was collected and heated at 95°C for 20 min, and subsequently assessed for its toxicity against *H. armigera* (Lone et al., 2016).

## 3 Results

### 3.1 *Bacillus thuringiensis* phenotypic characterization

**Figure 1:**
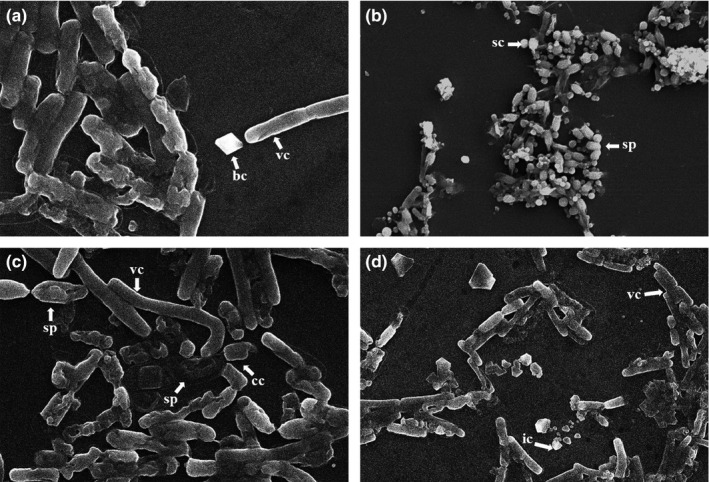
Scanning electron micrographs of *B. thuringiensis* cells showing presence of crystals, spores, and vegetative cells. (a) bipyramidal crystal (bc), (b) spherical crystal (sc), (c) cuboidal crystal (cc), (d) irregular crystal (ic). sp and vc indicate spores and vegetative cells, respectively

Eighty‐nine *Bacillus* strains were isolated by applying acetate‐penicillin selection methodology to 207 soil samples collected from 10 different regions representing three different environments of northwestern Himalayas (Table 1). Fifty‐six isolates were found to synthesize crystal protein during sporulation by phase contrast microscopy, the parasporal crystals were differentiated from the endospores by appearing as dark particles in contrast to endospores which appeared bright. Parasporal crystals were classified into four morphological classes based on SEM observations (Fig. 1); bipyramidal, spherical, cuboidal, and irregular types. *B. thuringiensis* was successfully isolated from all types of samples, recovery was the highest from forest soils (0.76) followed by plain agricultural soils (0.62) and the least from lake sediments (0.25) (Table 1). The average *Bt* index of the study was 0.62 (Table 1), (*Bt* index: ratio between number of *B. thuringiensis* colonies and total number of sporulating colonies examined).

### 3.2 Molecular characterization

The collection of *B. thuringiensis* isolates was characterized by assessing the number and type of *cry* and or *cyt* genes (PCR analysis) and the number and size of Cry and/or Cyt protein bands (SDS‐PAGE analysis).

#### 3.2.1 *cry/cyt* gene content

**Figure 2:**
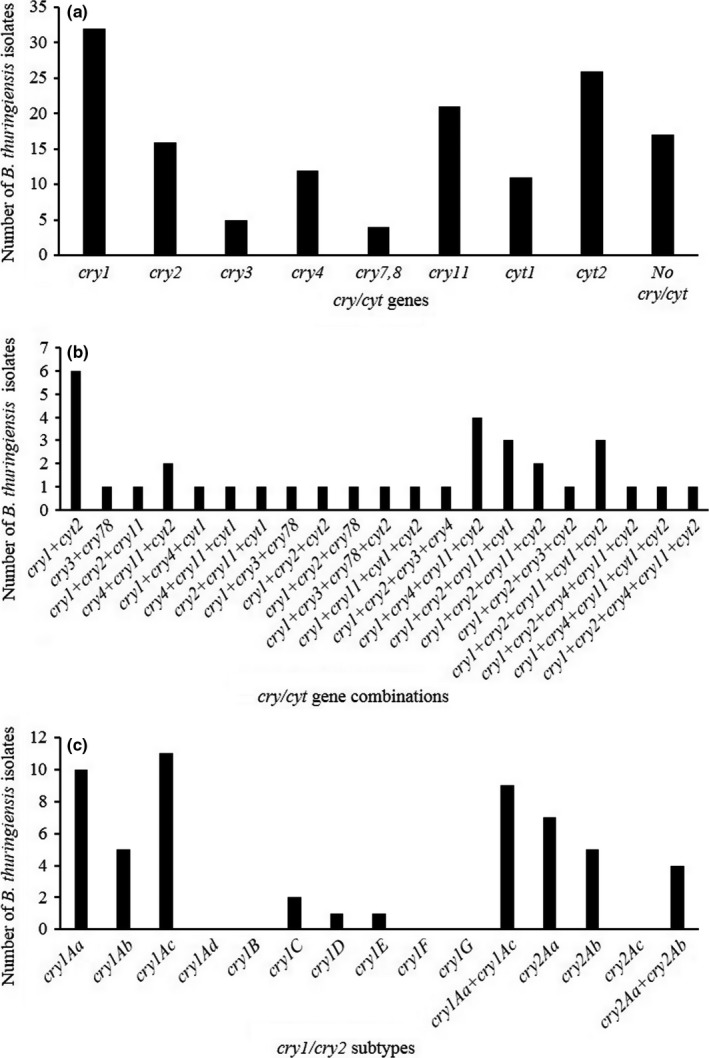
Occurrence rate of *cry* and *cyt‐*type gene combinations in the *B. thuringiensis* strains of western Himalayas. (a) Distribution of strains harboring single *cry*/*cyt* gene, (b) distribution of isolates harboring combination of two or more *cry*/*cyt* genes, (c) distribution of *cry*1 and *cry*2 subtype gene combinations among *cry*1 and *cry*2 harboring isolates

PCR screening using eight pairs of specific primers was performed to detect seven *cry* and two *cyt* gene families in our collection (Table S1). Primers for the detection of *cry*1, *cry*2, *cry*3, *cry*4, *cry*7,8, *cry*11, *cyt*1, *cyt*2 showed successful amplification as indicated by their specific product sizes of 1500, 701, 589, 439, 420, 445, 477, and 355 bp, respectively, on agarose gel electrophoresis (data not shown). Thirty‐nine isolates of the collection were found to be positive for the presence of *cry*/*cyt* genes tested, harboring either single or combination of these genes. Overall in our collection, isolates containing *cry*1 were the most abundant (57.1%) followed by *cyt*2 (46.42%), *cry*11 (37.5%), *cry*2 (28.57%), *cry*4 (21.42%), *cyt*1 (19.64%), *cry*3 (8.9%), *cry*7, 8 (7.14%), and 30.35% of the isolates did not react with any of the primers tested (Figure 2a). Four (10.24%) of the isolates carried single *cry*/*cyt* gene, whereas 7 (17.94%) isolates had combination of two, 9 (23.07%) isolates had combination of three, 13 (33.33%) isolates had combination of four and 6 (15.38%) isolates harbored combination of five *cry*/*cyt* genes (Figure 2b). The isolates harboring single gene showed equal dominance of *cry*1 and *cyt*2 (5.12% each), all the remaining genes were found in combination with other *cry*/*cyt* genes. Isolates harboring two genes showed two different combinations consisting of *cry*1 +  *cyt*2 (15.38%) and *cry*3 +  *cry*7, 8 (2.56%) (Figure 2b, Table 2). Isolates harboring three genes showed eight different combinations consisting of *cry*1 +  *cry*2 +  *cry*11 (2.56%), *cry*4 +  *cry*11 +  *cyt*2 (5.12%), *cry*1 +  *cry*4 +  *cyt*1 (2.56%), *cry*4 +  *cry*11 +  *cyt*1 (2.56%), *cry*2 +  *cry*11 +  *cyt*1 (2.56%), *cry*1 +  *cry*3 +  *cry*7, 8 (2.56%), *cry*1 +  *cry*2 +  *cyt*2 (2.56%), *cry*1 +  *cry*2 +  *cry*7, 8 (2.56%); within the group, *cry*1 is common in five of the eight combinations, while as, *cry*2 and *cry*11 is common in four combinations (Figure 2b, Table 2). Isolates harboring four genes showed seven different combinations consisting of *cry*1 +  *cry*3 +  *cry*7, 8 +  *cyt*2 (2.56%), *cry*1 +  *cry*11 +  *cyt*1 +  *cyt*2 (2.56%), *cry*1 +  *cry*2 +  *cry*3 +  *cry*4 (2.56%), *cry*1 +  *cry*4 +  *cry*11 +  *cyt*2 (10.25%), *cry*1 +  *cry*2 +  *cry*11 +  *cyt*1 (7.69%), *cry*1 +  *cry*2 +  *cry*11 +  *cyt*2 (5.12%), *cry*1 +  *cry*2 +  *cry*3 +  *cyt*2 (2.56%); within the group *cry*1 was found in all the combinations, *cyt*2 was found in five of the seven combinations and *cry*2, *cry*11 in four combinations (Fig. 2b, Table 2). Isolates harboring five genes showed four different combinations consisting of *cry*1 +  *cry*2 +  *cry*11 +  *cyt*1 +  *cyt*2 (7.69%), *cry*1 +  *cry*2 +  *cry*4 +  *cry*11 +  *cyt*2 (5.12%), *cry*1 +  *cry*4 +  *cry*11 +  *cyt*1 +  *cyt*2 (5.12%), *cry*1 +  *cry*2 +  *cry*4 +  *cry*11 +  *cyt*2 (5.12%); within the group *cry*1, *cry*11, and *cyt*2 were found in all the combinations, *cry*2 and *cry*4 in three of the four combinations (Fig. 2b; Table 2).

**Table 2:** Crystalline protein and gene content of *B. thuringiensis* isolates

Isolates	Crystal shape^a^	*cry* gene profiles	Major protein bands (kDa)
JK3	Bc, Sc, Ic, Tc	*cry*1C, *cry*3, *cry*7,8, *cyt*2	55, 65, 130
JK7	Bc, Sc, Ic, Tc	*cry*1Aa, *cry*1Ac, *cry*2Aa, *cry*2Ab *cry*11, *cyt*1, *cyt*2	55, 65, 70, 130
JK9	Bc, Sc, Tc	*cry*1Aa, *cry*1Ab, *cry*2Aa, *cry*2Ab *cry*11, *cyt*1, *cyt*2	55, 65, 70, 130
JK10	Tc	*cyt*2	25, 28, 35
JK11	Tc	*cyt*2	25, 28, 35
JK12	Bc, Sc, Tc	*cry*1Aa, *cry*1Ac, *cry*4, *cry*11, *cyt*2	55, 65, 130
JK14	Bc	*cry*1	65, 130
JK16	Sc, Tc	*cry*1, *cry*2, *cry*11, *cyt*1, *cyt*2	65, 70, 130
JK18	Sc, Ic, Tc	*cry*1Ab, *cry*4, *cry*11, *cyt*1, *cyt*2	65, 130
JK19	Bc, Sc	*cry*1Aa, *cry*1Ac, *cry*2Aa, *cry*11,	65, 70, 130
JK20	Sc	*cry*4, *cry*11, *cyt*2	NA
JK21	Sc	*cry*1, *cry*4, *cyt*1,	65, 130
JK22	Bc	*cry*1Ac, *cyt*2	65, 130
JK26	Bc	*cry*1Ab, *cyt*2	65, 130
JK32	Sc	*cry*4, *cry*11, *cyt*1	NA
JK33	Bc, Sc	*cry*1Aa, *cry*1Ac, *cry*1C, *cry*4, *cry*11, *cyt*2	65, 130
JK34	Bc	*cry*1Ab, *cry*1E	65, 130
JK36	Sc	*cry*2, *cry*11, *cyt*1	70, 65
JK37	Bc	*cry*1Aa, *cry*1Ac, *cry*1D, *cyt*2	65, 130
JK38	Bc	*cry*1, *cyt*2	65, 130
JK40	Bp, Tc	*cry*1, *cry*11, *cyt*1, *cyt*2	65, 130
JK48	Bp, Sc, Ic	*cry*1Aa, *cry*1Ac, *cry*2Ab, *cry*3, *cry*4	65, 70, 130
JK53	Sc, Tc	*cry*1Ac, *cry*4, *cry*11, *cyt*2	65, 130
JK55	Bc, Sc	*cry*1, *cry*2, *cry*11, *cyt*1	65, 70, 130
JK56	Bc	*cry*1, *cyt*2	65, 130
JK58	Sc	*cry*2Aa, *cry*2Ab, *cry*11, *cyt*1	65, 70
JK60	Ic, Tc	*cry*3, *cry*7,8	70
JK61	Ic, Tc	*cry*3, *cry*4, *cry*11, *cyt*2	70
JK63	Bc	*cry*1, *cyt*2	65, 70
JK64	Sc	*cry*1Ab, *cry*2, *cry*11, *cyt*2	65, 70, 130
JK65	Bc, Sc	*cry*1Aa, *cry*1Ac, *cry*2, *cry*3, *cyt*2	65, 70, 130
JK66	Sc	*cry*2Aa, *cry*11, *cyt*1	70
JK67	Sc	*cry*1, *cry*4, *cry*11, *cyt*2	65, 130
JK72	Bc	*cry*1Aa, *cry*1Ac, *cry*2Aa, *cry*11, *cyt*2	65, 70, 130
JK73	Bc, Ic	*cry*1, *cry*3, *cry*7,8	65, 70, 130
JK78	Sc	*cry*1, *cry*4, *cry*11, *cyt*2	65, 130
JK79	Sc	*cry*1, *cry*2, *cyt*2	65, 70, 130
JK82	Sc	*cry*4, *cry*11, *cyt*2	NA
JK86	Bc, Ic	*cry*1, *cry*2Aa, *cry*2Ab, *cry*7,8	65, 70, 130

a Bc, Sc, Ic, Tc indicate bipyramidal, spherical, irregular, and tiny crystal shapes (too small to record their shape), respectively.

^b^NA indicates that no major band was observed.

Isolates harboring *cry*1 and *cry*2 genes were further characterized by detection of the secondary rank of these genes using several sets of primers (Table S1). Both *cry*1 and *cry*2 harboring isolates showed the presence of different subtype genes. Two isolates showed the combination of three *cry*1, 10 isolates had combination of two *cry*1 and five isolates showed the presence of only one *cry*1‐subtype gene. *cry*1Ac was the most abundant constituting 34.3%, followed by *cry*1Aa (31.25%), *cry*1Ab (15.62%), *cry*1C (6.25%), *cry*1D (3.12%), and *cry*1E (3.12%) of the *cry*1‐positive isolates (Figure 2c). Among the *cry*2‐subtypes genes tested, *cry*2Aa was the most predominant, observed in 43.75%, followed by *cry*2Ab observed in 31.25% of the *cry*2‐positive isolates (Figure 2c). Four *cry*1 subtype genes, *cry*1Ad, *cry*1B, *cry*1F, *cry*1G, and one *cry*2 subtype, *cry*2Ac were not amplified in any of the *Bt* isolates with the sets of primers used in this study (Figure 2c).

#### 3.2.2 Protein profiling

**Figure 3:**
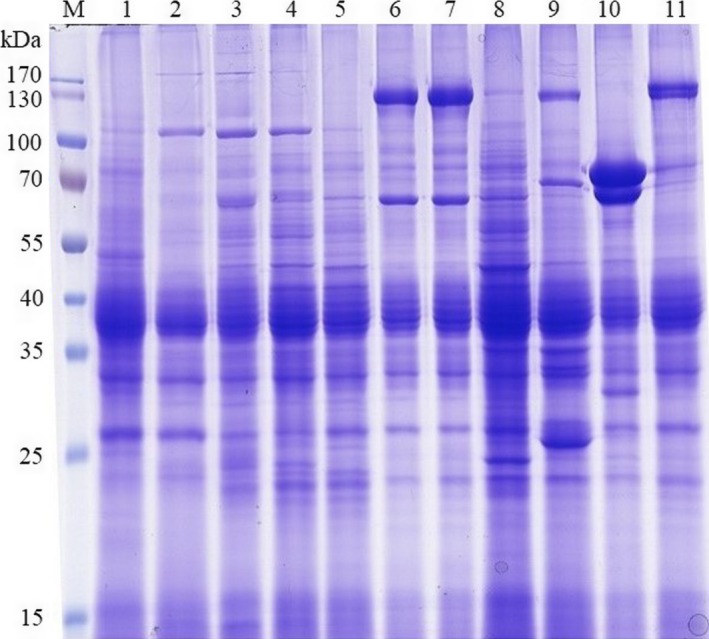
SDS‐PAGE of spore‐crystal mixture of *B. thuringiensis* strains showing diverse protein profiles. Lanes: M, prestained molecular mass marker (Thermo Scientific, USA); 1, 5, 8, no major proteins; 2, 3, 4, major protein of 110 kDa; 6, 7, 9, major proteins of 130 kDa and 65 kDa; 10, major protein of 70 kDa; 11, *Bt* kurstaki (4D1) showing major protein of 130 kDa

SDS‐PAGE analysis revealed great diversity with respect to the number and size of protein bands observed (Figure 3), even strains from the same sample showed diverse protein profiles (Table 2). Several strains in the collection showed identical protein profiles, such strains were considered as duplicate and only one strain per profile and sample was selected. The protein profiles of the isolated strains were compared to that of *B. thuringiensis* 4D1, the protein profiles are summarized in Table 2. The most common protein profile exhibited several polypeptides ranging in molecular mass from 25 kDa to 130 kDa (Figure 3). Most of the strains (42.8%) showed a protein composition resembling that of Lepidoptera‐toxic *B. thuringiensis* subsp. *kurstaki* consisting of two major protein bands of 130 kDa and 65 kDa, approximately (Figure 3 Lanes 6,7,11). A total of 28.5% of the isolates showed the presence of major polypeptide of 65–70 kDa resembling Diptera toxic *B. thuringiensis* subsp. *israelensis* (Figure 3 Lane 10), whereas 14.28% isolates represented a unique profile consisting of major polypeptides of 110 kDa and 30 kDa (Fig. 3 Lanes 2‐4). The majority of the isolates showed the presence of a number of polypeptides of approximately 70 kDa (Figure 3) and 35–55 kDa. Two polypeptides between 25 and 35 kDa were consistently observed in all the *B. thuringiensis* isolates including the reference strain.

### 3.3 Insect bioassay

**Figure 4:**
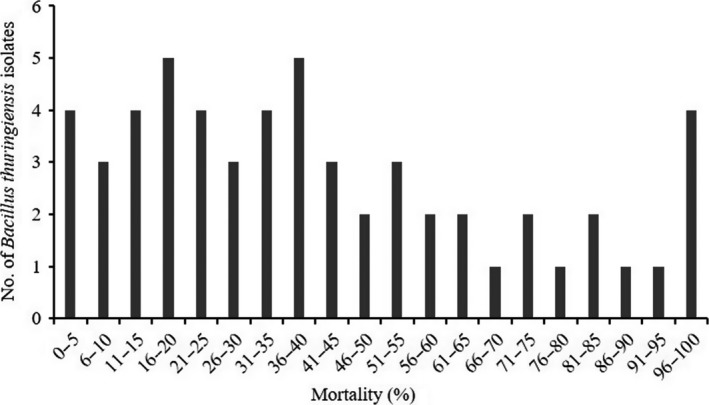
Mortality of *H armigera* larvae fed with diets containing highly concentrated spore‐crystal mixtures (1,000 μg ml^−1^) of different *B. thuringiensis* isolates

Toxicity screening against *H. armigera* was conducted in parallel to the PCR analysis, therefore, all the 56 *B. thuringiensis* strains of our collection were subjected to bioassays. Concentrated spore‐crystal suspensions (1,000 μg ml^−1^) were used for initial toxicity screening. Barring four isolates (JK12, JK22, JK48, JK72), which killed 96%–100% of *Helicoverpa* larvae within 7 days, preliminary screening revealed different levels of toxicity among the isolates with mortality ranging from 0% to 100% (Figure 4). These potentially toxic isolates were subjected to additional bioassays to determine the LC_50_ concentrations, using *B. thuringiensis kurstaki* (HD1) as positive control. The LC_50_ values of native isolates ranged from 184.62 to 259.93 μg ml^−1^, whreas that of HD1 was 195.32 μg ml^−1^. The negative control assays did not show any mortality. Although LC_50_ of all the native isolates was comparable to that of HD1, JK12 was found to be the most potent, showing the least LC_50_ (184.62 μg ml^−1^) compared to all the native isolates and to the positive control (HD1) as well (Table 3). All four toxic isolates and the *Bt* HD1 strain showed a drastic (~100%) reduction in mortality following heat treatment, which indicates that the toxic component is heat‐labile high molecular mass fraction and not the heat‐resistant β‐exotoxin.

**Table 3:** Toxicity of spore‐crystal mixtures of *Bt* isolates toward *H. armigera*

Strain tested	Slope ± SE	LC 50 (μg ml^−1^)	95% Fiducial limits (μg ml^−1^)	Toxicity index^a^
JK12	1.72 ± 1.66	184.62	151.99–220.65	1.05
JK22	1.68 ± 0.54	275.39	179.49–434.47	0.70
JK48	1.62 ± 0.72	256.29	158.02–419.69	0.76
JK72	1.72 ± 0.92	259.93	217.68–310.68	0.75
HD1	1.33 ± 1.64	195.32	158.80–236.49	1.00

a Toxicity index indicates the relative toxicity of native to reference strain. It is the ratio of LC_50_ of HD1 to that of the tested strain; the larger the ratio, the higher the toxicity.

## 4 Discussion

In the *Bacillus cereus* group, the presence of a parasporal inclusion body is the single most discriminatory criterion for the identification of isolates in the species *B. thuringiensis* (Soufiane, Baizet, & Cote, 2013). Based on the presence of parasporal crystals, 56 out of 89 *Bacilli* strains were classified as *B. thuringiensis*. The results revealed that *B. thuringiensis* was ubiquitously distributed in the soils of northwestern Himalayas tested, with no history of *Bt* application. However, the *Bt* index (the estimation of the success of *Bt* isolation) varied in samples collected from different regions. The average *Bt* index recorded in the study was 0.62, which means that 62% of the sporulating colonies examined showed the presence of crystals. The average is higher compared to some previous isolation programs (less than 0.1 to 0.52) (Bravo et al., 1998; Chak, Tsen, & Yamamoto, 1994; Vidal‐Quist, Castañera, & González‐Cabrera, 2009), lower compared to that observed (0.85) by Martin and Travers (1989) and similar to that observed (0.6 to 0.7) by Obeidat, Hassawi, and Ghabeish (2004). Lake sediments showed the least *Bt* index (0.25), whereas forest soils showed the highest *Bt* index (0.76) (Table 1). It is difficult to assess the likely reason for the differences in *Bt* index values, but the sampling size, difference in geomorphology of the region, complex interaction between the bacterium and its insect host could influence their numbers in a particular environment. The predominance of bipyramidal and round crystal shapes observed in this study corroborates with the previous reports (Jara, Maduell, & Orduz, 2006; Vidal‐Quist et al., 2009).

PCR‐based identification of *cry* genes was introduced by Carozzi, Kramer, Warren, Evola, and Koziel (1991) to predict insecticidal activity of *B. thuringiensis* strains and has become popular tool for the large‐scale screening of *B. thuringiensis* collections for the presence of insecticidal genes. In this study, different *cry* and *cyt* gene profiles have been detected, the prevalence of *cry*1 was found to be the most abundant followed by *cyt*2, *cry*11, *cry*2, *cry*4, *cyt*1, *cry*3, and *cry*7, 8. The frequency of genes observed was in accordance with the frequency reported by Porcar and Juarez‐Perez (2003); Bravo et al. (1998); Baig and Mehnaz (2010), all of whom reported the predominance of *cry*1 genes, and differed from that observed by Ben‐Dov et al. (1997); Pinto and Fiuza (2003); Baig et al. (2010); and Mendoza, Portillo, Arias, Ribas, and Olmos (2012), who reported the predominance of *cry*9, *cry*2, *cry*4, and *cry*4 genes, respectively. These data indicate the difference in the diversity of different crystalline protein coding genes in *B. thuringiensis* of different geographic regions. Only four isolates showed the presence of single gene, two each harboring *cry*1 and *cyt*2, all remaining positive strains showed presence of *cry/cyt* genes in combinations (Figure 2b). The most frequent combinations observed consisted of lepidopteran‐specific *cry*1 with dipteran‐specific *cry*2, *cry*4, *cry*11, and *cyt*2 genes, less frequent combinations included lepidopteran‐specific *cry*1 with dipteran‐specific *cry*2, *cry*4, and coleopteran‐specific *cry*3 genes (Figure 2b). Strains containing presence of intergroup crystalline protein‐coding genes are ideal candidates for the development of broad‐spectrum biocontrol agents. Other researchers have reported different combinations of *cry* genes in the same *Bt* strains, *cry*3 and *cry*7 or *cry*9 (Aronson, 1993; Ben‐Dov et al., 1997), *cry*1, *cry*3A, *cry*3B, and *cry*7A (Bravo et al., 1998). The presence of more than one *cry* and or *cyt* genes in these isolates suggests that they have high frequency of genetic information exchange. Among the genes identified in this study, *cry*1, *cry*2, encode lepidopteran‐specific toxins; *cry*2, *cry*4, *cry*11, *cyt*1, *cyt*2, encode dipteran‐specific toxins, and *cry*3, *cry*7, 8 encode coleopteran‐specific toxins. As a result, 33, 26, and 6 strains of our collection are at least theoretically toxic to lepidopteran, dipteran, and coleopteran pest, respectively.

Among the *cry*1 gene subtypes tested in this study, *cry*1Ac was the most dominant, present in (34.37%) followed by *cry*1Aa (31.25%) and *cry*1Ab (15.62%) of the *cry*1‐harboring isolates. *cry*1Ad, *cry*1B, *cry*1F, *cry*1G genes were not observed in any of the *cry*1‐harboring isolates and 46.87% of the *cry*1 gene‐harboring isolates did not show amplification of any *cry*1 subtypes tested suggesting that these isolates may contain other *cry*1 subtype genes not tested in this study. Various reports have shown a high frequency of one or the other subtype of *cry*1 genes, like, Chak et al. (1994) reported abundance of *cry*1A genes, whereas Arrieta, Hernandez, and Espinoza (2004) reported abundance of *cry*1D, and Baig et al. (2010) reported high frequency of *cry*1K subtypes. The *cry*1 gene‐harboring isolates showed the presence of different combinations of *cry*1 subtype genes with *cry*1Aa‐*cry*1Ac combination constituting 28.15% of the *cry*1 gene‐harboring isolates (Figure 2c). Two of the isolates JK33 and JK37 had unique combinations of *cry*1Aa, *cry*1Ac, *cry*1C, and *cry*1Aa, *cry*1Ac, *cry*1E, respectively (Table 2).

Among the *cry*2 subtypes tested, the combination of *cry*2Aa‐*cry*2Ab was the most frequent followed by *cry*2Aa and *cry*2Ab alone, whereas *cry*2Ac was not observed in any of the *cry*2‐positive isolates (Figure 2c), the similar pattern of distribution was observed in soils of Argentina (Sauka, Cozzi, & Benintende, 2005) and China (Liang et al., 2011). However, Ben‐Dov et al. (1997) observed highest frequency of *cry*2Ab alone followed by *cry*2Aa‐*cry*2Ab and *cry*2Ab‐*cry*2Ac in *Bt* isolates in samples from Israel, Kazakhstan, and Uzbekistan. The prevalence of *cry*2Aa‐*cry*2Ab gene combination, as observed in this study may be commonly found in nature but its biological significance is yet to be studied. Of the 17 isolates that did not react with any of the primers tested, many showed presence of crystals on microscopy. Although such isolates did not show appreciable toxicity against *H. armigera*, the presence of crystals in these isolates suggests that they may harbor insecticidal proteins specific to some other target insect. Previous studies have reported varying number of isolates producing no PCR product (ranging from 14% to 40%) from different geographical regions of the world (Bravo et al., 1998; Uribe, Martinez, & Ceron, 2003; Vilas‐Bôas & Lemos, 2004). All these results indicate the presence of numerous isolates harboring novel and unidentified crystalline protein genes throughout the world. The results presented here demonstrate that PCR‐based approach can be used for systematic, large‐scale screening of *B. thuringiensis* isolates to identify known *cry*/*cyt* genes and to characterize their toxicity.

SDS‐PAGE profiles of the sporulated *Bt* strains revealed great diversity in our collection (Figure 3, Table 2), the diverse profiles observed are in accordance with the earlier reports (Chak et al., 1994; Jara et al., 2006) and lower compared to others (Iriarte, Porcar, Lecadet, & Caballero, 2000; Vidal‐Quist et al., 2009). Correspondence of SDS‐PAGE profiles to the *cry*/*cyt* gene profiles detected by PCR, however, has not been straightforward in all the strains. The most likely reasons for this difference are: the strains tested could produce endotoxins whose genes are not tested in this study, the protein bands observed could be either protoxins, activated toxins, or proteolysis intermediates, the same trend has been observed earlier (de Barros Moreira & Silva‐Filha, 2007; Mohan & Gujar, 2003). The most prominent molecular mass polypeptides (130 kDa, 65 kDa) observed correspond to the Cry1 and Cry2 proteins, respectively (Armengol, Escobar, Maldonado, & Orduz, 2007; Seifinejad, Salehi Jouzani, Hosseinzadeh, & Abdmishani, 2008). The lower molecular mass polypeptides (35–55 kDa) observed match with the Cyt proteins or digestion products of many Cry proteins (Gough, Kemp, Akhurst, Pearson, & Kongsuwan, 2005).

Various bioassay methods like diet incorporation (Dulmage, Dulmage, Boening, Rehnborg, & Hansen, 1971), surface contamination (Ignoffo, 1965), and droplet feeding (Hughes & Wood, 1981) have been used to obtain lethal concentration (LC) estimates. The first two methods are suitable for post‐first instar larvae as they are able to fully consume small blocks of food, whereas the third method is suitable for neonates as their transparent body makes it easy to track the colored feeding solution in their gut. In this study, diet incorporation method was used against second instar larvae of *H. armigera*, the earlier instar larvae were used because the older larvae tend to be more tolerant to *Bt*‐based pesticides (Sanahuja, Banakar, Twyman, Capell, & Christou, 2011). The preliminary toxicity screening of *Bt* isolates using higher doses of spore‐crystal suspensions (1,000 μg ml^−1^) revealed that these strains exhibit a wide range of toxicity (causing 0%–100% mortality) toward second instar larvae of *H. armigera* (Figure 4). Twenty‐seven percent of the isolates causing mortality of 60% or above were considered as toxic, among them, four isolates causing (96%–100%) mortality were considered to be highly toxic and were subjected to detailed toxicity analysis to determine their LC_50_ values. The LC_50_ values of native isolates observed were in the range of 184.62 to 259.93 μg ml^−1^ in comparison to HD1 which showed LC_50_ of 195.32 μg ml^−1^ (Table 3).

As the strain JK12 was actually most toxic to *H. armigera* than any other strain tested in this study, there is a possibility of synergism between the two different lepidopteran‐specific (*cry*1Aa and *cry*1Ac) Cry proteins observed in this strain by PCR analysis. It must, however, be noted that mere presence of more than one *cry* genes does not account for additive effect in toxicity, as all genes may not be expressed simultaneously or even if expressed, they may act antagonistically by competing for the same binding site in insect gut as reported earlier (Liao, Heckel, & Akhurst, 2002; Morse, Yamamoto, & Stroud, 2001). Previous studies have reported pronounced differences in the toxicity response of *H. armigera* to *B. thuringiensis* insecticidal proteins (Avilla, Vargas‐Osuna, González‐Cabrera, Ferré, & González‐Zamora, 2005; Bird & Akhurst, 2007; Liao et al., 2002). These differences could be result of variation in insecticidal genes present, differences in insect strain used and difference in the diet composition or bioassay method employed (Avilla et al., 2005; Bird & Akhurst, 2007). Therefore, comparison of the toxicity data in different studies must be based on the relative toxicity rather than the absolute values of toxicity estimates. Moreover, in order to have more realistic estimates of toxicity of a particular strain, many independent populations of susceptible insect should be tested, preferably with the same protocols.

*B. thuringiensis* presents great diversity with respect to their crystalline protein content and insecticidal activity even in the isolates from same soil sample. The diversity and activity might have relationship with the geographical location of the samples. An important characteristic observed in this study was the presence of more than one *cry/cyt* genes in many of the isolates, with differences, at least in theory, in terms of their host range. Moreover, the presence of *cry/cyt* genes from different groups were also observed in the same strain, suggesting that some of these strains (particularly *Bt* JK12) after proper evaluation against different insect species can be developed into broad‐spectrum biopesticides. Seventeen of the *Bt* isolates in the collection did not interact with the eight pairs of primers tested, and were also not toxic to *H. armigera*. However, presence of crystals in these isolates suggests that they may harbor novel *cry/cyt* genes not tested in this study, hence, their toxicity toward other insect species needs to be evaluated. The results of this study confirm the significance of continuous exploration of new *Bt* stains from different ecological regions of the world.

## Conflict of Interest

The authors declare no conflict of interest.
