# Supplementary material for: Selection and characterization of Bacillus thuringiensis strains from northwestern Himalayas toxic against Helicoverpa armigera
Source: Microbiologyopen. 2017 Oct 18;6(6):e00484. doi: 10.1002/mbo3.484 (PMC5727364; doi:10.1002/mbo3.484)
Supplement: Supplementary file 1 [file MBO3-6-na-s001.doc]

***Table S1*** *Characteristics of primers and respective reference strains used to detect cry and cyt type genes*

| **Primer Pair** | **Sequence (5’ to 3’)** | **Gene recognized** | **AT^2^ (°C)** | **Product (bp)** | **Source** | **Control strain** |
| --- | --- | --- | --- | --- | --- | --- |
| Family 1 | (+)20 mer 5’- MDATYTCTAKRTCTTGACTA-3’  (-) 20 mer 5’-TRACRHTDDBDGTATTAGAT-3’ | *cry*1 | 45 | 1,500 | Juarez-Perez  et al. 1997 | BGSC 4D1 |
| IA’s | (+) 20 mer 5’-CAATAGTCGTTATAATGATT-3’ | *cryIA* | 47 | 1,720 | -do- |  |
| IAa | (+) 20 mer 5’-TTCCCTTTATTTGGGAATGC-3’ | *cryIAa* | 47 | 1,286 | -do- |  |
| IAb | (+) 20 mer 5’-CGGATGCTCATAGAGGAGAA-3’ | *cryIAb* | 47 | 1,371 | -do- |  |
| IAc | (+) 20 mer 5’-GGAAACTTTCTTTTTAATGG-3’ | *cryIAc* | 47 | 844 | -do- |  |
| IAd | (+) 20 mer 5’-ACCCGTACTGATCTCAACTA-3’ | *cryIAd* | 47 | 1,212 | -do- |  |
| IB | (+) 20 mer 5’-GGCTACCAATACTTCTATTA-3’ | *cryIB* | 47 | 1,323 | -do- |  |
| IC | (+) 20 mer 5’-ATTTAATTTACGTGGTGTTG-3’ | *cryIC* | 47 | 1,176 | -do- |  |
| ID | (+) 20 mer 5’-CAGGCCTTGACAATTCAAAT-3’ | *cryID* | 47 | 1,138 | -do- |  |
| IE | (+) 20 mer 5’-TAGGGATAAATGTAGTACAG-3’ | *cryIE* | 47 | 1,137 | -do- |  |
| IF | (+) 20 mer 5’-GATTTCAGGAAGTGATTCAT-3’ | *cryIF* | 47 | 967 | -do- |  |
| IG | (+) 20 mer 5’-GGTTCTCAAAGATCCGTGTA-3’ | *cryIG* | 47 | 1,128 | -do- |  |
| Un2 | (+) 25 mer 5’-GTTATTCTTAATGCAGATGAATGGG-3’  (–) 25 mer 5’-CGGATAAAATAATCTGGGAAATAGT-3’ | *cry*2 | 59 | 701 | Ben-Dov et al. 1997 | BGSC 4J3 |
| EE-2Aa(r) | (–) 21mer 5’- GAGATTAGTCGCCCCTATGAG-3’ | *cry2Aa1* | 59 | 498 | -do- |  |
| EE-2Ab(r) | (–) 25 mer 5’-TGGCGTTAACAATGGGGGGAGAAAT-3’ | *cry2Ab2* | 59 | 546 | -do- |  |
| EE-2Ac(r) | (–) 24 mer 5’-CGTTGCTAATAGTCCCAACAACA-3’ | *cry2Ac* | 59 | 725 | -do- |  |
| Un3 | (+)26 mer 5’-CGTTATCGCAGAGAGATGACATTAAC-3’  (-) 23 mer 5’ CATCTGTTGTTTCTGGAGGCAAT -3’ | *cry*3 | 59 | 589 | -do- | BGSC 4AA1 |
| Un4 | (+) 24 mer 5’-GCATATGATGTAGCGAAACAAGCC- 3’  (–) 25 mer 5’-GCGTGACATACCCATTTCCAGGTCC- 3’ | *cry*4 | 59 | 439 | -do- | BGSC 4Q1 |
| Un7,8 | (+) 22 mer 5’-AAGCAGTGAATGCCTTGTTTAC-3’  (–) 19 mer 5’-CTTCTAAACTTGACTACTT-3’ | *cry*7*, cry*8 | 54 | 420,423 | -do- | BGSC 4AA1 |
| EE-11A | (+) 20 mer 5’- CCGAACCTACTATTGCGCCA-3’  (–) 20 mer 5’-CTCCCTGCTAGGATTCCGTC-3’ | *cry11A1* | 59 | 445 | -do- | BGSC 4Q1 |
| UNcyt1 | (+) 24 mer 5’-CCTCAATCAACAGCAAGGGTTATT-3’  (–) 27 mer 5’-TGCAAACAGGACATTGTATGTGTAATT-3’ | *cyt1* | 52 | 477 | Ibarra et al. 2003 | BGSC 4Q1 |
| UNcyt2 | (+) 24 mer 5’-ATTACAAATTGCAAATGGTATTCC-3’  (– ) 28 mer 5’-TTCAACATCCACAGTAATTTCAAATGC-3’ | *cyt2* | 50 | 355 | -do- | BGSC 4Q1 |

**^2^** annealing temperature
